# Genome-wide identification of the AcMADS-box family and functional validation of *AcMADS32* involved in carotenoid biosynthesis in *Actinidia*

**DOI:** 10.3389/fpls.2023.1159942

**Published:** 2023-06-19

**Authors:** Zhiyi Lin, Zunzhen He, Daoling Ye, Honghong Deng, Lijin Lin, Jin Wang, Xiulan Lv, Qunxian Deng, Xian Luo, Dong Liang, Hui Xia

**Affiliations:** College of Horticulture, Sichuan Agricultural University, Chengdu, China

**Keywords:** kiwifruit, MADS-box, expression profile, carotenoids, transgenic plants

## Abstract

MADS-box is a large transcription factor family in plants and plays a crucial role in various plant developmental processes; however, it has not been systematically analyzed in kiwifruit. In the present study, 74 AcMADS genes were identified in the Red5 kiwifruit genome, including 17 type-I and 57 type-II members according to the conserved domains. The AcMADS genes were randomly distributed across 25 chromosomes and were predicted to be mostly located in the nucleus. A total of 33 fragmental duplications were detected in the AcMADS genes, which might be the main force driving the family expansion. Many hormone-associated *cis*-acting elements were detected in the promoter region. Expression profile analysis showed that *AcMADS* members had tissue specificity and different responses to dark, low temperature, drought, and salt stress. Two genes in the AG group, *AcMADS32* and *AcMADS48*, had high expression levels during fruit development, and the role of *AcMADS32* was further verified by stable overexpression in kiwifruit seedlings. The content of α-carotene and the ratio of zeaxanthin/β-carotene was increased in transgenic kiwifruit seedlings, and the expression level of *AcBCH1/2* was significantly increased, suggesting that *AcMADS32* plays an important role in regulating carotenoid accumulation. These results have enriched our understanding of the MADS-box gene family and laid a foundation for further research of the functions of its members during kiwifruit development.

## 1 Introduction

MADS-box genes encode one of the largest transcription factor (TF) families in plants and play a vital role in various aspects of plant development, especially in flower organogenesis (Messenguy and Dubois, 2003; Hassankhah et al, 2020). MADS-box TFs contain a highly conserved MADS-box DNA-binding domain, which is composed of 56–58 amino acids in the N-terminal region that bind to CArG boxes (CC(A/T)_6_GG), regulating the transcription of downstream genes (Becker and Theissen, 2003). Plant MADS-box genes have been subdivided into two main types: type I and type II (Alvarez-Buylla et al., 2000). Type I contains 1 or 2 exons, encoding an SRF-like MADS domain, and has been further subdivided into three groups: Mα, Mβ, and Mγ. Type II TFs generally contain six introns and seven exons, encoding four domains: MADS, I, K, and a C-terminal domain (Henschel et al., 2002). The first exon encodes an MEF2-like or MIKC type MADS domain; therefore, type II genes have been divided into Mδ/MIKC^*^ and MIKC^c^ groups (Kaufmann et al., 2005). The MIKC^c^ genes have been further divided into 14 subgroups based on their phylogeny.

Current studies on MADS mainly focused on exploring the regulatory role of MIKC^c^-type genes in plant growth and development, involving almost all related aspects, such as floral organ formation, fruit development and maturation, root and leaf development (Henschel et al., 2002; Kaufmann et al., 2005; Li et al., 2019), *etc*. In the ‘ABCDE’ model of floral organ development, most floral organ characteristic genes belong to the MIKC^c^-type, which participate in the regulation of the homologous transformation of floral organs (Saedler et al., 2001; Becker and Theissen, 2003). In respiratory climacteric fruit tomato, *SlMBP8* inhibits tomato fruit maturation by negatively regulating ethylene biosynthesis (Yin et al., 2017), while *RIN* and *FUL1*/*FUL2* act as global regulators of ripening, affecting the climacteric rise of ethylene, pigmentation changes, and fruit softening, and regulating carotenoid synthesis (Shima et al., 2014; Stanley and Yuan, 2019). In sweet orange (*Citrus sinensis*), *CsMADS5* and *CsMADS6*, homologues of *FRUITFULL* and *SlTAGL1*, have been shown to upregulate carotenoid biosynthesis by activating the transcription of *PSY*, *PDS*, and *LCYb1* (Lu et al., 2018; Lu et al., 2021). Recently, *MdMADS6* has been identified in apple to be involved in the regulation of carotenoid biosynthesis in fruits (Li et al., 2022). In *Arabidopsis thaliana*, at least seven members of MADS are preferentially expressed in roots, and the MIKC^c^ subfamily gene *ANR1* is almost exclusively expressed in the roots (Burgeff et al., 2002) and the MIKC^c^ subfamily gene *AGL14* regulates auxin transport during root growth and development in *Arabidopsis* (Garay-Arroyo et al., 2013).

In recent years, MADS-domain proteins have been identified in *Arabidopsis* (Parenicová et al., 2003), tomato (Wang et al., 2019), rice (Arora et al., 2007), soybean (Shu et al., 2013), apple (Tian et al., 2015), and pear (Wang et al., 2017). However, the MADS-box family has not been systematically analyzed in kiwifruit. Kiwifruit is one of the most successful domesticated fruit trees in modern times and its fruit is rich in vitamin C (Huang et al., 2013). In kiwifruit, only four MADS-box genes in the SVP subgroup have been identified, including *SVP1, SVP2, SVP3*, and *SVP4. SVP1–4* have high expression levels in bud tissue and play an important regulatory role in flower bud differentiation and flowering time (Wu et al., 2012; Wu et al., 2014; Wu et al., 2017). In this study, the MADS-box gene family was comprehensively identified in the kiwifruit genome and the expression profiles of its members were analyzed under different abiotic stresses and during fruit development. In addition, the regulatory function of *AcMADS32* in carotenoid biosynthesis was verified through stable overexpression in kiwifruit. The results provide a comprehensive understanding of the MADS-box gene family in kiwifruit and have laid a foundation for further research on the functions of its members during the development of kiwifruit.

## 2 Materials and methods

### 2.1 Plant material and treatment

Mature leaves, roots, stems, and flowers were collected from three 10-year-old kiwifruit trees (*Actinidia chinensis* var. *chinensis* ‘Donghong’) in Pujiang County (103°51’ E, 30°42’ N), Chengdu, China. Fruit samples during development were collected at 60, 90, and 150 days after anthesis (Liang et al., 2020). At least 18 fruits were collected each time, and six were mixed as a replication. All samples were prepared for three biological replicates. Samples were transported to the laboratory as soon as possible and stored at −80°C for RNA extraction.

The sterile tissue culture seedlings of ‘Jinyan’ (*A. chinensis* × *A. eriantha*, a yellow-fleshed kiwifruit cultivar bred in China) were used for stress treatments, referring to our previous study (Xia et al., 2022). Sixty 4-month-old seedlings with ten leaves were equally divided into five groups for treatments, including the control (CK), dark treatment (DK), low-temperature treatment (LT), PEG-simulated drought treatment (DR), and salt treatment (ST). Except for the LT group growing at 4 ± 2°C, the other four treatment groups were all cultured at 25 ± 2°C, light/dark 16 h/8 h. The DK group was shaded with black cloth, the DR group was treated with 40% (w/v) PEG-6000 to simulate drought stress, and the ST group was treated with 100 mmol/L NaCl. After 48 h treatment, the leaves of every four seedlings were sampled and mixed as one replicate, repeated three times. Samples were stored at −80°C immediately after being flash-frozen in liquid nitrogen.

### 2.2 Identification of MADS gene family members in kiwifruit

DNA sequence, CDS sequence, protein sequence, and gff3 files of the ‘Red5’ kiwifruit genome were downloaded from the Kiwifruit Genome Database (www.kiwifruitgenome.org). The protein sequences of 107 *Arabidopsis* MADS were downloaded from the TAIR website (www.arabidopsis.org/). The domain sequence (HMM model file) of MADS transcription factor (code: PF00319) was downloaded from the Pfam website (http://pfam.xfam.org/) as the seed file to search MADS proteins in the kiwifruit genome file (set e-value at 0.01) by running a hidden Markov model (HMM) search. The searched proteins were compared with *Arabidopsis* MADS using multiple BLAST; incomplete and repeated sequences were deleted, and the remainder were identified as AcMADS genes.

### 2.3 Physical and chemical property analysis and subcellular localization

Online software ExPASy (https://web.expasy.org/protparam/) was used to predict the number of amino acids, molecular weight, and isoelectric point. Wolf PSort (https://wolfpsort.hgc.jp/) was used to predict the subcellular localization of the MADS protein sequences.

### 2.4 Chromosome localization

The location information of MADS genes on the kiwifruit chromosome was extracted from the gff3 file and submitted to the MG2C (http://mg2c.iask.in/mg2c_v2.0/) website to draw chromosome location maps.

### 2.5 Gene structure and conserved motif analysis

The distribution information of introns and exons of AcMADS genes was obtained from the gff3 file of the kiwifruit genome and submitted to the Gene Structure Display Server (http://gsds.gao-lab.org/) to draw the gene structure map. The online software MEME (http://meme-suite.org/tools/meme) was used to search for conserved motifs.

### 2.6 Phylogenetic analysis

After multiple sequence alignment of the MADS proteins of kiwifruit and *Arabidopsis* by ClustalW, a phylogenetic tree was constructed using the neighbor-joining (NJ) method with the following parameters: p-distance model, paired deletion, bootstrap 1,000, and other parameters to default.

### 2.7 Intragroup collinearity analysis

Collinearity relationships within the kiwifruit genome were analyzed using MCScanX (Wang et al., 2012) with default parameters. Pairs of collateral homologous genes were screened according to the following criteria: the sequence similarity of two genes was greater than 75% with more than 75% alignment length of the longer one. Circos (Krzywinski et al., 2009) was used to visualize the relationship between the chromosomal location of the MADS genes, and KaKs_Calculator 2.0 (Wang et al., 2010) was used to calculate the Ka/Ks value of MADS paralogues.

### 2.8 Putative cis-regulatory elements analysis

The upstream 1,500 bp promoter sequences of the AcMADS start codons were extracted from the kiwifruit genome data file and submitted to PlantCARE (http://www.dna.affrc.go.jp/PLACE/signalscan.html) for prediction analysis of *cis*-acting elements.

### 2.9 Gene expression analysis by qRT-PCR

Total RNA was extracted and quantified using a NanoPhotometer^®^ spectrophotometer (Implen, Westlake Village, CA, USA). A 1 μg aliquot of extracted RNA was reverse transcribed into cDNA using a PrimeScript™ RT reagent kit with gDNA Eraser (Perfect Real Time) (Takara, Dalian, China). The primers were designed using Primer Premier5.0 and are listed in **Table S1**. qRT-PCR was performed with a CFX96 instrument (Bio-Rad, CA, USA) using a SYBR Premix Ex Taq Kit (Takara, Dalian, China) with the following parameters: 95°C for 10 s, followed by 40 cycles of 95°C for 10 s and 60°C for 30 s. Three technical and biological replicates were established for each reaction. The relative expression level was calculated using the 2^-△△ Ct^ method (Livak and Schmittgen, 2001).

### 2.10 Generation of transgenic kiwifruit

Sterile kiwifruit seedlings (*A. eriantha* cv. White) maintained in our laboratory were used for stable transformation and were subcultured on Murashige and Skoog (MS) medium supplemented with 1.0 mg L^-1^ 6-benzylaminopurine (6-BA) and 0.1 mg L^-1^ naphthaleneacetic acid (NAA).

The leaves were precultured on MS medium containing 3.0 mg·L^−1^ zeatin (ZT) and 0.1 mg·L^−1^ NAA for 3 days and transferred to *Agrobacterium* suspension containing recombinant plasmid PBI121-35S-AcMADS32 for infection for 10 min. They were co-cultivated in the dark on MS medium consisting of 100 μmol·L^−1^ acetosyringone, 3.0 mg·L^−1^ ZT, and 0.1 mg·L^−1^ NAA for 2 days. Afterward, the explants were inoculated into MS medium supplemented with 20 mg·L^−1^ kanamycin and 200 mg·L^−1^ timentin to induce callus information. After 4 weeks, the calluses with bud spots were transferred into MS medium supplemented with 2.0 mg·L^−1^ 6-BA, 0.1 mg·L^−1^ NAA, 20 mg·L^−1^ kanamycin, and 200 mg·L^−1^ timentin and cultured under a light intensity of 90 μmol·m^−2^·s^−1^, a photoperiod of 16 h light/8 h dark, a temperature of 25 ± 2°C, and a relative humidity of 75%.

### 2.11 Determination of carotenoid content in the transgenic lines by HPLC

Carotenoid content was determined using the HPLC method described previously (Xia et al., 2022). Briefly, an Agilent 1260 HPLC system (Agilent, Santa Clara, USA) equipped with a VWD detector and a YMC C30 column (250 mm × 4.6 mm, 5 μm) were used. Solvent A (30% methyl tertbutyl ether) and solvent B (70% methanol) were used as the mobile phase, with a flow rate of 0.5 ml/min, a column temperature of 25°C, and a detection wavelength of 450 nm.

## 3 Results

### 3.1 Identification and chromosome localization of MADS-box genes in kiwifruit

**Figure 1:**
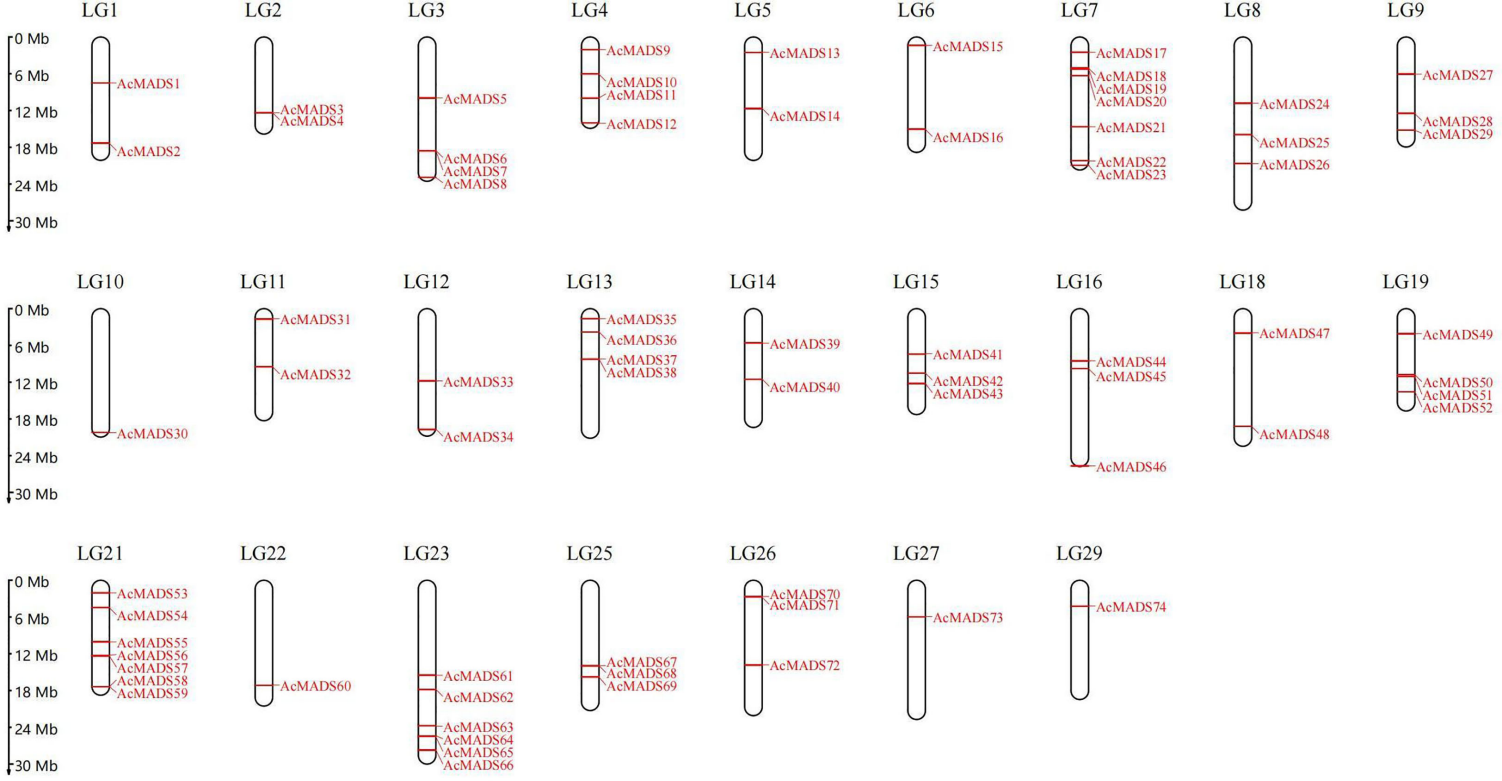
Chromosome locations of the identified AcMADS genes.

A total of 80 genes were initially predicted as MADS genes. After removing redundant protein sequences and confirming the integrity of conserved domains, 74 MADS genes were identified (**Table S2**). These genes were randomly distributed on 25 chromosomes (**Figure 1**), among which Chr7 and Chr21 were the most with seven, Chr23 with six, and Chr10, Chr22, Chr27, and Chr29 with only one, and the number of *MADS* genes on the other 19 chromosomes ranged from two to four. According to their position on chromosomes, they were named *AcMADS1–74*.

### 3.2 Physicochemical properties and subcellular localization of AcMADS gene family members

The basic information of AcMADS, including amino acids, molecular weight, isoelectric point, instability, and the fat solubility index were analyzed by using ExPASY (listed in **Table S2**). The lengths of the amino acid sequences of 74 MADS-box proteins varied greatly, ranging from 137 to 499 aa, with the corresponding molecular weights ranging from 15.32 to 56.40 kDa, with protein isoelectric points of 4.59–10.2. Based on GRAVY analysis, 52 AcMADS proteins were alkaline, accounting for 70.3%, and all AcMADS proteins were hydrophilic. Subcellular localization prediction found that 57 AcMADS genes were most likely localized in the nucleus, accounting for 77%, and seven were most likely located in the cytoplasm. *AcMADS1*, *AcMADS20*, *AcMADS43*, *AcMADS56*, and *AcMADS63* had the highest probability of localizing in mitochondria. *AcMADS8*, *AcMADS10*, *AcMADS17*, *AcMADS54*, and *AcMADS73* are most likely located in the chloroplast (**Table S2**).

### 3.3 Gene structures and conserved motif analysis in AcMADS-box genes

**Figure 2:**
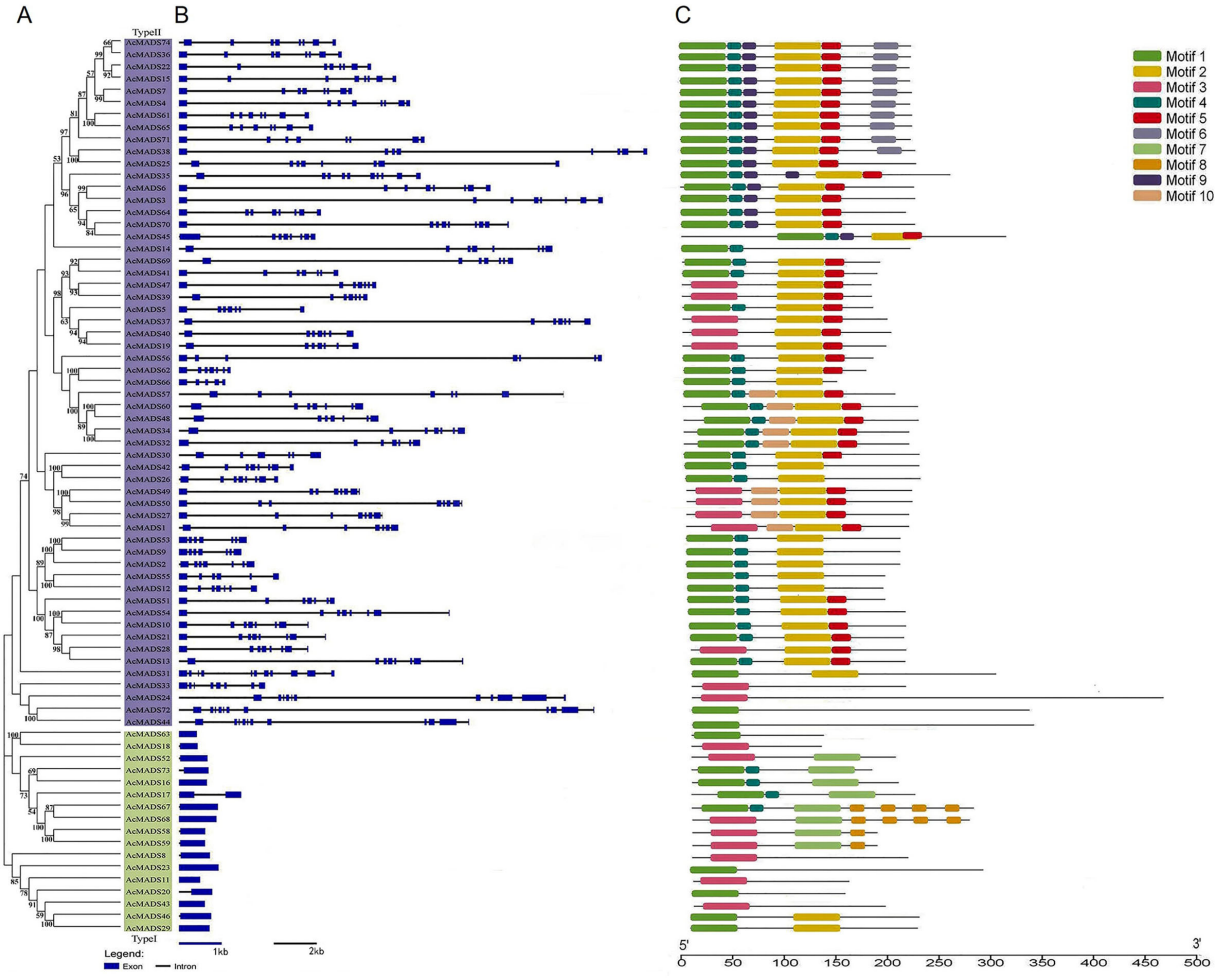
**(A–C)** Evolutionary analysis of kiwifruit MADS proteins **(A)**, gene structure **(B)**, and conserved motifs **(C)**.

A phylogenetic tree was constructed using the AcMADS protein sequences (**Figure 2A**) and gene structure was analyzed (**Figure 2B**). A total of 74 AcMADS-box genes were mainly divided into two categories: 57 type-II *MADSs* (in purple) and 17 type-I *MADS* (in green). The number of exons ranged from one to 11. Almost all type-I AcMADS genes contained only one exon, except *AcMADS17*, which contained two exons. Most type-II *AcMADS* genes contained seven to eight exons, except *AcMADS24*, *AcMADS44*, and *AcMADS72*, which had 11 exons.

The conserved motifs of AcMADS were predicted using the online software MEME; 10 motifs were detected (**Figure 2C**; **Table S3**). Motif1 and motif3 were MADS domains and motif4 and motif5 were K-box domains. Almost all AcMADS proteins contained the MADS domain. In addition to AcMADS24 and AcMADS72, type-II AcMADS proteins contained the K-box domain, whereas type-I AcMADS proteins contained only the MADS domain.

### 3.4 Phylogenetic analysis of AcMADS genes

**Figure 3:**
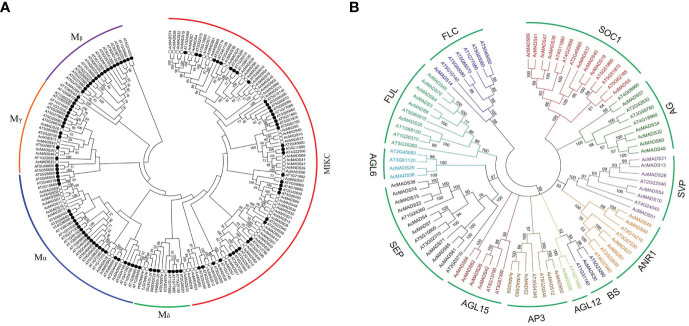
Phylogenetic analysis of the MADS-box gene family in kiwifruit. **(A)** Phylogenetic tree of MADS-box proteins in *Actinidia chinensis* (white dot) and *Arabidopsis thaliana* (black dot), with red as *AcMADS* and black as *AtMADS*. **(B)** Phylogenetic analysis of the MIKC gene family in *Actinidia chinensis* and *Arabidopsis thaliana*.

A phylogenetic tree of MADS proteins in kiwifruit and *A. thaliana* was constructed using the NJ method. A total of 17 type-I genes could be further subdivided into three subclasses: Mα, Mβ, and Mγ, containing 10, one, and six members, respectively (**Figure 3A**). A total of 57 type-II genes could be divided into two subfamilies, Mδ/MIKC^*^ (five members) and MIKC^c^, which could be further subdivided into 12 groups: SEP, AGL6, FUL, SOC1, AGL15, AP3/PI, AG, SVP, ANR1, AGL12, BS, and FLC (**Figure 3B**).

### 3.5 Collinearity analysis of AcMADS genes

**Figure 4:**
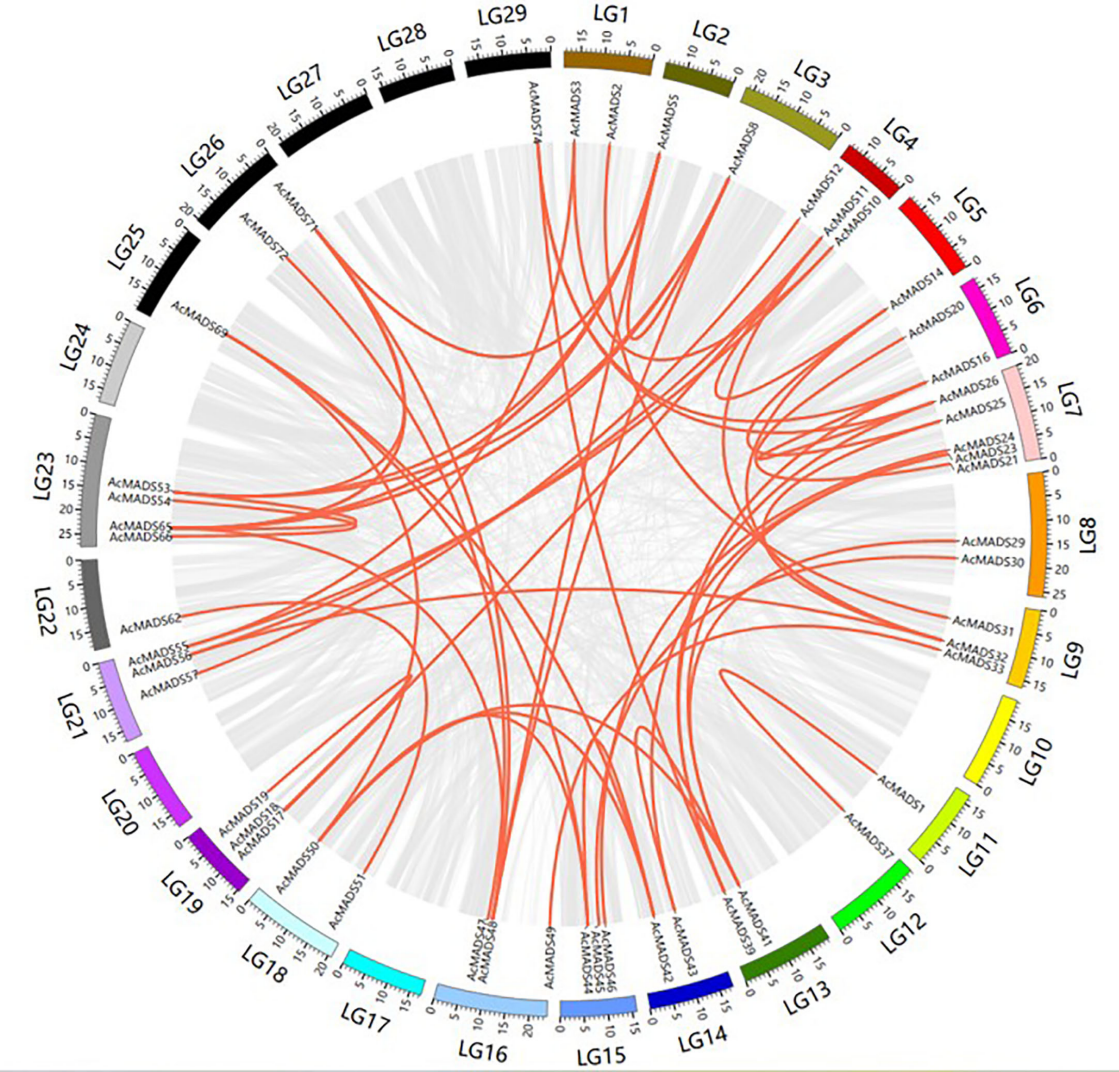
Collinearity analysis and gene duplication of AcMADS-box in kiwifruit.

To identify duplication events in AcMADS genes, a collinearity analysis was performed using MCScanX software (**Figure 4**; **Table S4**). A total of 33 segmental duplications were detected, which might be the main force driving the expansion of the MADS gene family.

Ka/Ks was widely used to detect whether genes had been subjected to selection pressure during evolution. The results showed that Ka/Ks of most collinearity pairs was less than 1, indicating that these genes might have undergone strong purifying selection during evolution. The Ka/Ks value of *AcMADS19*/*AcMADS37* was greater than 1, suggesting that the genes might have experienced positive selection (**Table S4**).

### 3.6 Analysis of the cis-acting elements of AcMADS-box genes

**Figure 5:**
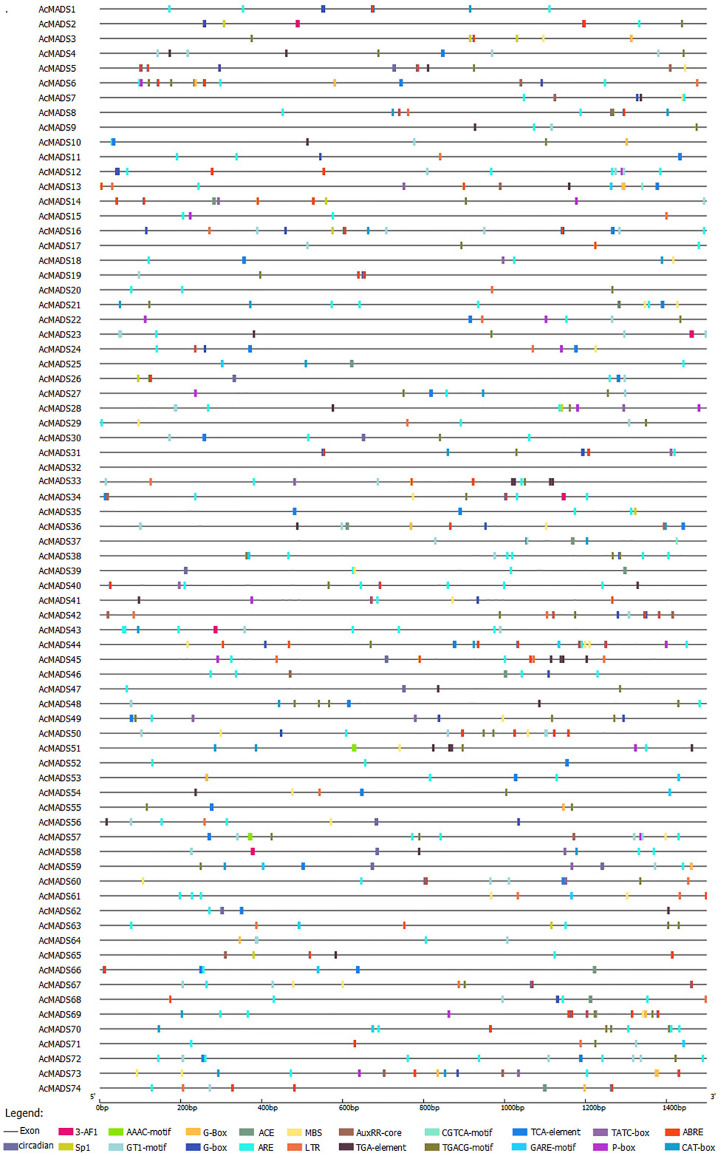
Collinearity analysis and gene duplication of AcMADS-box in kiwifruit.

To further explore the function of MADS genes in kiwifruit, the *cis*-elements of gene promoters were predicted using the PlantCARE website. Light signal-related elements were detected in 65 of 74 MADS gene promoters, among which *AcMADS15* had the most with 10. All *AcMADS*s except *AcMADS28* had hormone-associated *cis*-acting elements, such as those responding to auxin, abscisic acid, gibberellin, salicylic acid, and methyl jasmonate. In addition, abiotic stress-related response elements, such as drought and low temperature, were also detected (**Figure 5**; **Table S5**).

### 3.7 Expression profiles of AcMADS genes in different tissues

**Figure 6:**
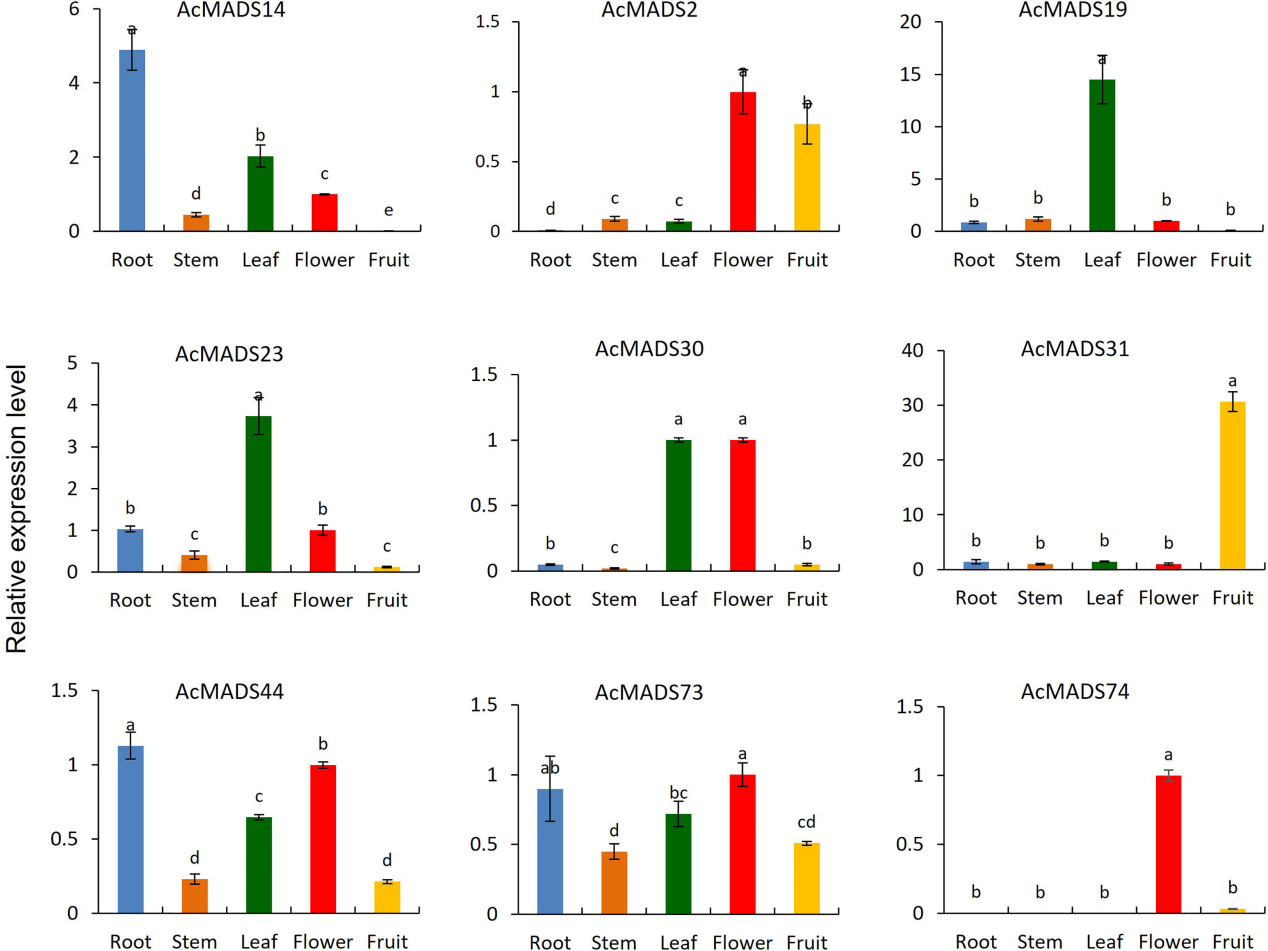
The tissue-specific expression of AcMADS genes. Data are mean ± standard error (n=3), with different letters indicating significant differences among treatments (*P*<0.05).

Nine AcMADS genes from different groups were randomly selected for expression analysis in the leaves, stems, roots, and fruits of the kiwifruit by qRT-PCR (**Figure 6**). The tissue-specific expression characteristics of these genes were different. *AcMADS14* was highly specifically expressed in roots. *AcMADS19* and *AcMADS23* were highly expressed in leaves. Only *AcMADS31* was highly expressed in fruit, while *AcMADS2*, *AcMADS30*, and *AcMADS74* were highly expressed in flowers. However, there was no highly specific expression of AcMADS in stems. These results suggest that different AcMADS members may be functionally differentiated during evolution, thus influencing different aspects of plant development.

### 3.8 Expression profiles of AcMADS genes under various abiotic stresses

**Figure 7:**
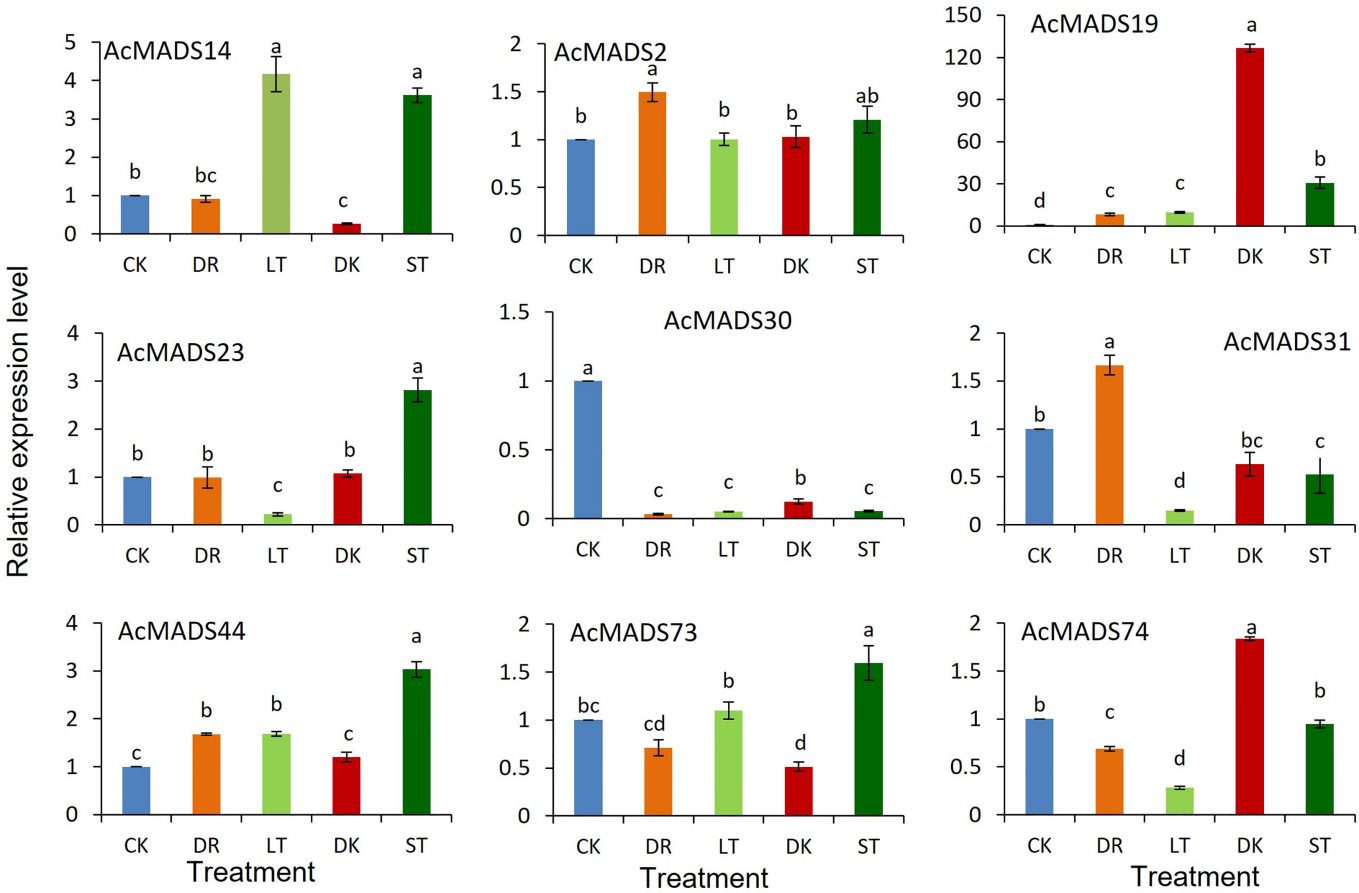
Relative expression levels of AcMADS genes under different stresses. CK, control; DK, dark; LT, low temperature; DR, drought; ST, salt. Data are mean ± standard error (n=3), with different letters indicating significant differences among treatments (*P*<0.05).

The expression profiles of AcMADS genes were detected under darkness, low temperature, drought, and salt stress (**Figure 7**). *AcMADS19* and *AcMADS74* were induced to be highly expressed in response to drought stress. With low-temperature treatment, *AcMADS14*, *AcMADS19*, and *AcMADS44* were upregulated, and the expression of *AcMADS14* was increased by fourfold, while *AcMADS23*, *AcMADS31*, and *AcMADS74* were downregulated. Under dark treatment, AcMADS19 expression dramatically increased over 120 times compared with the control. Under salt stress, *AcMADS14*, *AcMADS19*, *AcMADS23*, *AcMADS44*, and *AcMADS73* were upregulated, while *AcMADS30* and *AcMADS31* were downregulated.

### 3.9 Expression of AcMADS genes at different fruit growth stages

**Figure 8:**
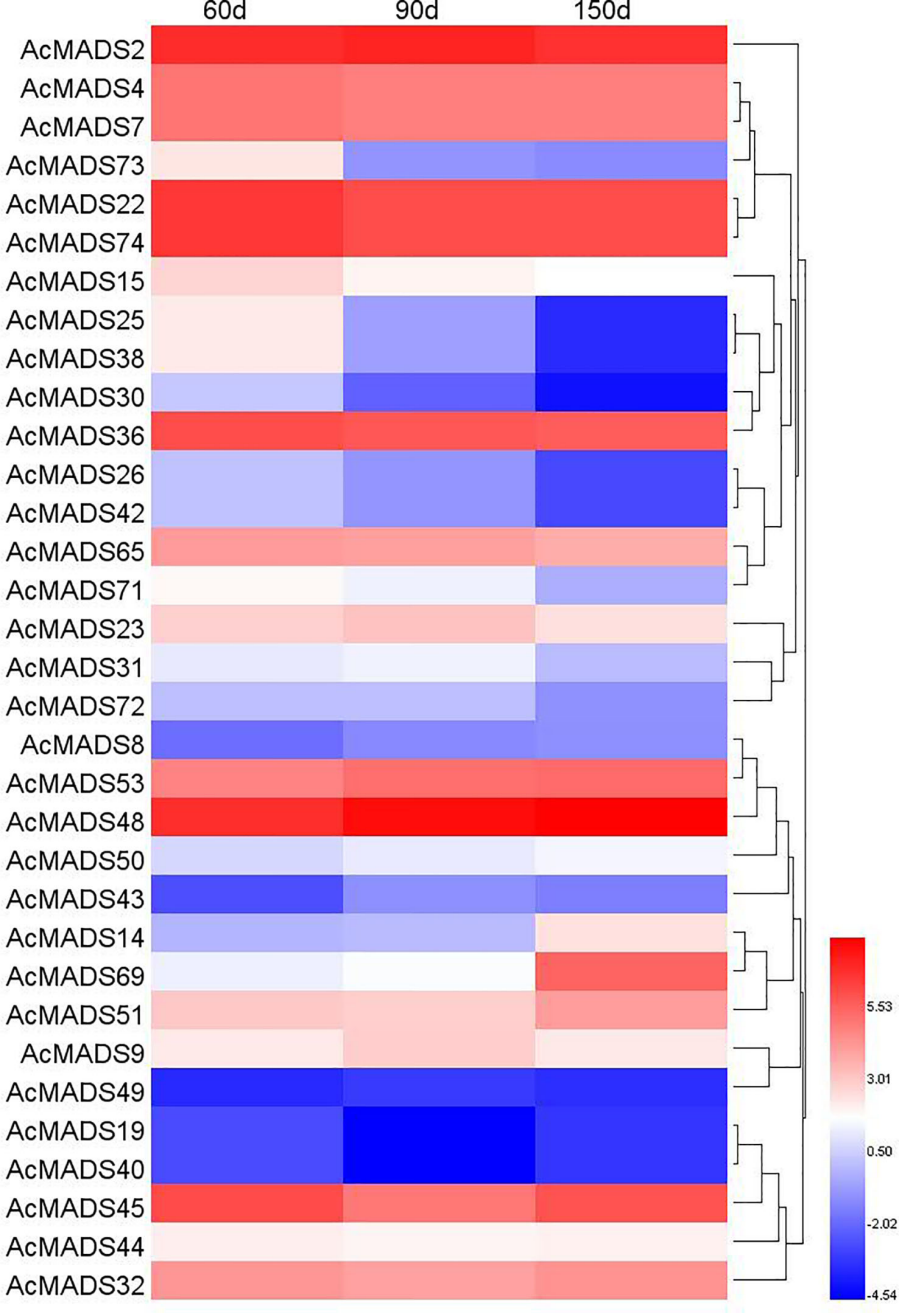
Expression heatmap of AcMADSs at different fruit growth stages in kiwifruit.

It has been reported that MADS-box genes play an important role in the regulation of fruit development and ripening (Karlova et al., 2014); therefore, the expression profiles of AcMADS genes during fruit development were analyzed based on our previous RNA-seq data on ‘Donghong’ kiwifruit (Liang et al., 2020). A total of 33 *AcMADS*s were expressed during fruit development, including four type-I genes, *AcMADS8*, *AcMADS23*, *AcMADS43* and *AcMADS73*, the expression levels of which were lower than those of the type-II genes (**Figure 8**).

Some type-II genes had relatively high expression levels and changed significantly at different stages, including *AcMADS32*, *AcMADS48* (AG group), *AcMADS2*, *AcMADS53* (AP3/PI group), *AcMADS51* (SVP group), *AcMADS4*, *AcMADS7*, *AcMADS22*, *AcMADS36*, *AcMADS65*, *AcMADS74* (SEP group), and *AcMADS45* (FUL group). The expression level of *AcMADS32* was higher at the young fruit stage (60 days) and fruit maturity stage (150 days), and the expression level of *AcMADS48* (AG) increased gradually with fruit growth. The expression levels of *AcMADS22* and *AcMADS74* (SEP) remained high throughout the development period. The expression pattern of *AcMADS51* in the SVP group was similar to that of *AcMADS32*.

### 3.10 Overexpression AcMADS32 in kiwifruit regulated the expression of carotenoid synthesis genes

**Figure 9:**
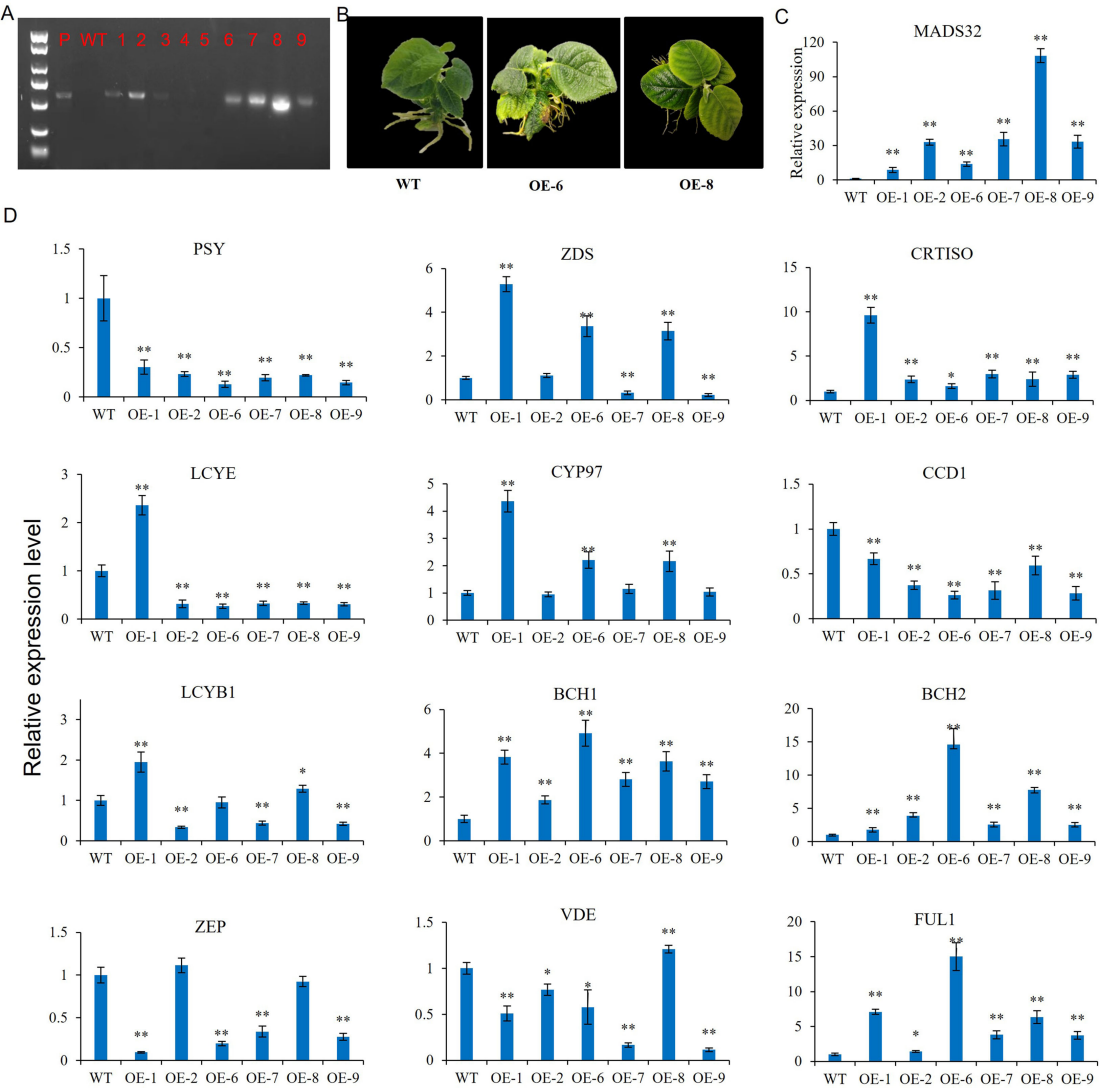
**(A–D)** *AcMADS32* overexpression in kiwifruit verified by PCR **(A)**, phenotype **(B)**, and the relative expression level of *AcMADS32* **(C)** and carotenoid biosynthetic genes **(D)**. Asterisks indicate significant differences from the wild type by Student’s *t*-test at *P*<0.05 (*) or *P*<0.01 (**).

Previous studies have shown that MADS in the AG group may be involved in the regulation of carotenoid accumulation (Stanley and Yuan, 2019). *AcMADS32* is a member of the AG group, and its expression pattern during fruit development was consistent with the change in carotenoid content in our previous study (Xia et al., 2021 and Xia et al., 2022); therefore, we selected *AcMADS32* and verified its role in carotenoid accumulation by overexpressing it in kiwifruit. Six positive strains were obtained through PCR amplification of DNA from 26 resistant buds (**Figure 9A**). The leaves of the transgenic kiwifruit plants were significantly more yellow than those of the wild type (**Figure 9B**). The expression level of *AcMADS32* was significantly higher than that of the wild type, and the expression of OE-8 was more than 100 times that of the wild type (**Figure 9C**). Carotenoid biosynthetic gene expression in wild type and the transgenic line was further examined by qRT-PCR (the carotenoid biosynthesis pathway is shown in **Supplementary Figure 1**). The results showed that the expression patterns of carotenoid pathway genes in transgenic strains were inconsistent (**Figure 9D**). *PSY*, *LCYE*, *ZEP*, and *CCD1* (in the carotenoid biosynthetic pathway as shown in **Supplemental Figure 1**) were significantly downregulated in almost all *AcMADS32*-overexpressing lines, while *CRTISO*, *BCH1*, *BCH2*, and *FUL1* were upregulated. *VDE* was downregulated in most transgenic lines, except for OE-8, in which it was upregulated. *ZDS* was upregulated in OE-1, OE-6, and OE-8, but down-regulated in OE-7 and OE-9. The expression pattern of *CYP97* and *LCYB1* in transgenic strains was similar to that of *ZDS*.

### 3.11 AcMADS32 overexpression modulated carotenoid content in transgenic kiwifruit leaves

**Figure 10:**
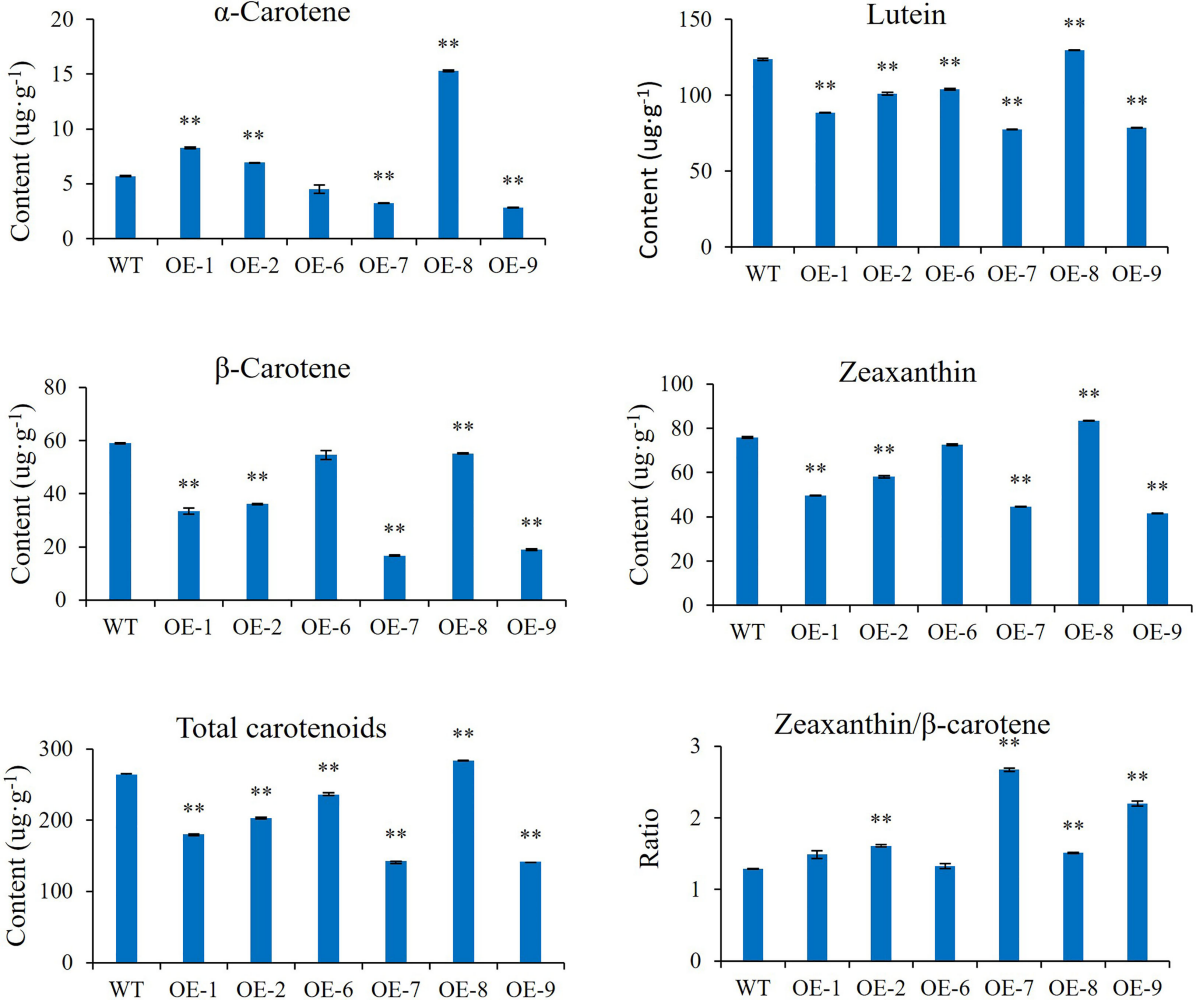
Carotenoid content in *AcMADS32* transgenic kiwifruit lines. Data are mean ± standard deviation (n=3). Asterisks indicate significant differences from the wild type by Student’s *t*-test at *P*<0.01 (**).

α-Carotene, lutein, β-carotene, and zeaxanthin were identified as the main carotenoid components in transgenic plant leaves by HPLC (**Figure 10**). Compared with the wild type, the lutein, zeaxanthin, and total carotenoid content in almost all transgenic lines decreased significantly, except for OE-8, which showed an increase. β-carotene content was significantly reduced in all transgenic lines. The content of α-carotene was increased in OE-1, OE-2, and OE-8 but decreased in OE-7 and OE-9. The ratio of zeaxanthin/β-carotene was increased in OE-2, OE-7, and OE-9.

## 4 Discussion

MADS-box genes encode a large TF family in plants, which plays a vital role in various aspects of plant development (Messenguy and Dubois, 2003). Therefore, a comprehensive understanding of the MADS family and the function of key members is meaningful for the modification of crop yield and quality. In recent years, the MADS family has been identified in many horticultural plants, such as pineapple (Zhang et al., 2020), apple (Tian et al., 2015), pear (Wang et al., 2017), tomato (Wang et al., 2019), iris (Bar-Lev et al., 2021), banana (Lakhwani et al., 2022), chrysanthemum (Won et al., 2021), pomegranate (Zhao et al., 2020), rhododendron (Huo et al., 2021), and litchi (Guan et al., 2021). A total of 74 *MADS* family members were identified in the kiwifruit genome in this study, which is smaller than that in *Arabidopsis* (106), poplar (105) and apple (147), and comparable with that of rice (75). In addition, the number of type-I members in kiwifruit decreased by two-thirds compared with *Arabidopsis*, while the number of type-II members expanded, which might have occurred as a result of recent duplications. Indeed, a large number (33 pairs) of fragmental duplication events were detected (**Table S4**), suggesting that most type-I members may have been lost under purifying selection during evolution, while type-II members were retained and replicated. Type-I MADS proteins hardly contain the K-box domain, and most type-II MADS proteins contain the K-box domain, which was consistent with the hypothesis that the K-box domain evolved after the differentiation of type-I genes (Becker and Theissen, 2003). The analysis of promoter *cis*-acting elements shows that most *cis-*acting elements are related to light and hormones, which may be related to MADS genes that regulate plant growth and development.

Temporal and spatial expression of genes is crucial for plant growth and development and provides important insights into gene function. According to the RNA-seq data (Liang et al., 2020), the expression profiles indicated that the expression patterns of the MADS-box genes were different during fruit development. A total of 33 MADS-box genes were detected at different fruit stages, and 3 AG-, 2 AP3-, 1 SVP-, and 6 SEP-group genes presented high transcript abundances in the fruit, which suggests that they may play critical roles in fruit development. Similarly, a MIKC-type MADS-box transcription factor, *PavAGL15*, is expressed at high levels in the flower buds, blossoms, and young fruit of sweet cherry and can bind the promoter of *PavCYP78A9* to control sweet cherry fruit size (Dong et al., 2022). In oil palm, real-time quantitative PCR results confirmed that the expression of *EgAGL9* increased rapidly during the last stages of oil palm mesocarp development (Zhang et al., 2022).

Four MADS-box genes, *AcMADS2*, *AcMADS30*, *AcMADS73*, and *AcMADS74*, exhibited higher expression levels in the flower of kiwifruit, indicating that they play an important role in the regulation of flower development. RNA-seq expression patterns of MADS-box genes in four different tissues revealed that more genes were highly expressed in the flowers of pineapple (Zhang et al., 2020), and the same situation was found in longan (Wang et al., 2022). Although most MADS-box genes have been reported to be involved in flower and fruit development, we observed high expression of *AcMADS13* and *AcMADS36* in the root and *AcMADS19* and *AcMADS49* in the leaves, suggesting that these MADS-box genes may play a critical role in regulating root or leaf development. A previous study observed 17 highly expressed MADS-box genes in the root of orchard grass, which indicated their critical roles in regulating root development (Yang et al., 2022). Studies have shown that *CiMADS43* can interact with *CiAGL9* and is involved in leaf development in citrus (Ye et al., 2021). *In situ* hybridization has shown that all type-II classic MADS-box genes in *Selaginella moellendorffii* have broad but distinct patterns of expression in vegetative and reproductive tissues (Ambrose et al., 2021). These results indicate the wide expression spectrums and versatile functions of MADS-box genes in different organs.

In recent years, some MADS genes have been proven to be important for the regulation of fruit ripening and carotenoid biosynthesis. The transient transformation of *MdMADS6* promoted carotenoid accumulation in apple fruit by acting on the downstream target genes *MdCCD1*, *MdPDS*, and *MdHYD* (Li et al., 2022). In citrus, *CsMADS6* directly regulates the expression of *LCYb1* and other carotenoid genes to affect carotenoid accumulation (Lu et al., 2018). Most notably, four members of the MADS family, *SlTAGL1*, *SlRIN*, *SlFUL1*, and *SlFUL2*, not only participate in the regulation of fruit ripening but also bind to multiple structural genes in the carotenoid biosynthesis pathway to stimulate their transcriptional activities (Fujisawa et al., 2013; Fujisawa et al., 2014; Stanley and Yuan, 2019). *AcMADS32* is a homologous gene to *SlTAGL* and may have similar functions; in this study, its function in carotenoid accumulation was verified by overexpressing it in kiwifruit.

In this study, *AcMADS32* overexpression significantly influenced carotenoid content and the expression profiles of carotenoid-related genes in transgenic plant leaves (**Figure 8**). *PSY* has been identified as an important flow-limiting enzyme in the carotenoid synthesis pathway (Bramley, 2002; Tao et al., 2007). In this study, the significant downregulation of *PSY* expression led to an overall decrease in the content of total carotenoids, α-carotene, lutein, β-carotene, and zeaxanthin in transgenic plants (**Figure 10**). This result was somewhat surprising because it was different from the result of *SlTAG* in tomatoes. *SlTAGL1* was a positive regulator of carotenoid accumulation in tomatoes. In RNAi plants of *SlTAGL1*, the expression of *PSY1* is downregulated and the expression of LYC-B and CYC-B is upregulated, resulting in a decrease in total carotenoid content, while β-carotene and lutein content is increased (Vrebalov et al., 2009). Although *PSY* is downregulated, most other structural genes are upregulated, particularly *BCH1* and *BCH2*. As a result, the ratio of zeaxanthin/β-carotene is increased in overexpressing plants, which is similar to the carotenoid accumulation in transgenic citrus calli (Lu et al., 2018). The upregulated expression level of *LCYe* and *CYP97* was the reason for the accumulation of lutein in the transgenic lines (**Figure 8**). *PSY* has generally been recognized as a key determinant of the total amount of carotenoids in several fruits, such as tomato (Bramley, 2002) and citrus (Tao et al., 2007); however, carotenoid levels in the leaves of *Arabidopsis AtPSY*-overexpressing lines remain unchanged (Lätari et al., 2015). These opposite results may be related to the fact that MADS-box in different tissues has different functions. On the other hand, while *PSY* was downregulated in the overexpression lines in this study, other downstream genes were upregulated, which may be another reason for the accumulation of zeaxanthin and lutein in the transgenic line. Additionally, the downregulated transcript level of *CCD1*, which catalyzes the enzymatic degradation of carotenoids to yield volatile compounds or other apocarotenoids, facilitates carotenoid accumulation in transgenic lines.

## 5 Conclusion

In this study, a total of 74 MADS-box genes were identified in the kiwifruit genome. The chromosomal distribution, conserved motifs, gene structures, and phylogenetic relationships of these AcMADS genes were characterized. Furthermore, the expression patterns of nine AcMADS genes were analyzed in different tissues during fruit development and under abiotic stress. In addition, *AcMADS32* was selected for functional study in the carotenoid biosynthesis process by transforming it into kiwifruit. *AcMADS32* overexpression regulated the expression of carotenoid-related genes and influenced the accumulation of carotenoid components. These results provide comprehensive basic data for further study on the function of the MADS-box genes in kiwifruit.

## Data availability statement

The original contributions presented in the study are included in the article/**Supplementary Material**. Further inquiries can be directed to the corresponding authors.

## Author contributions

HX and DL conceived the experiments. ZL, ZH, and DY carried out the experiments with the help of HD, JW, XLv, LL, XLu, and QD contributed the plant materials and data analysis. ZL and HX wrote the manuscript and DL edited the manuscript. All authors contributed to the article and approved the submitted version.
