# Supplementary material for: Genome-wide identification of the AcMADS-box family and functional validation of AcMADS32 involved in carotenoid biosynthesis in Actinidia
Source: Front Plant Sci. 2023 Jun 19;14:1159942. doi: 10.3389/fpls.2023.1159942 (PMC10315656; doi:10.3389/fpls.2023.1159942)
Supplement: Supplementary file 1 [file DataSheet_1.pdf]

Table S1 The primer sequences for qRT-PCR

Gene	Forward primer (5'→3')	Reverse primer (5'→3')
AcMADS2	GATCTGTGGAGCACGCACTA	GCGCAATTCATGGATGCTCA
AcMADS13	ATTCCCCACCAAATTTGACA	TTGCCATACACAGAGCTTGC
AcMADS14	TGAGTTGAAGCGGATCGAGG	TTTTTCTCAAGCTGTCGCCG
AcMADS19	ATAGAGAACGCCACGAGCAG	TGTGGTTCCGATAGCGTTGT
AcMADS25	CACATCCATCCGTGTTTCTG	ACCATTTCTTCGCTTCGAGA
AcMADS26	TTGAGTGTTTGGGGGAAAAG	GAGAATCGCAAGCTCTTTGG
AcMADS30	AGAGCACGATAACCGGGAAC	GCGGACTCAAGCTGTTGTTC
AcMADS31	GGAGAGAAGCAGATCGGAGC	AGCATCACCTTCGAAAACCCT
AcMADS32	AGGTTGAGCTGCAAAATGCT	TTTGGTCCTGGCGAGAATAC
AcMADS45	TGGCAGCAAAGAATGAACAG	GTTGCGGTGAAAGTGGTTTT
AcMADS49	AAACCACAGGCAACTTTTGG	TCCTTTCTCATTCGGACACC
AcMADS56	ACTCAACTGGGGGAACACAG	TCCGGGCATATACCTGAGAC
AcMADS74	ATCAACCGGCAAGTGACCTT	AGGGTTTTGAGCATGCTCGA

Table S2 The essential information of MADS-box gene family identified in kiwifruit

Gene Name	Gene locus	AA	MW (KDa)	IP	Instability index	Adipose index	hydropathicity	Subcellular localization	Group
AcMADS1	Acc00495	235	27.42	8.82	55.38	86.26	-0.807	mitochondrion	ANR1
AcMADS2	Acc01045	226	26.60	9.48	42.53	84.12	-0.799	cell nucleus	AP3
AcMADS3	Acc02284	248	28.50	9.82	66.47	83.75	-0.746	cell nucleus	FUL
AcMADS4	Acc02285	244	28.40	6.54	38.16	77.95	-0.747	cell nucleus	SEP
AcMADS5	Acc03527	201	23.22	5.87	63.82	88.26	-0.685	cell nucleus	SOC1
AcMADS6	Acc04040	247	28.58	9.27	61.67	81.66	-0.799	cell nucleus	FUL
AcMADS7	Acc04041	245	28.28	6.11	46.06	83.63	-0.717	cell nucleus	SEP
AcMADS8	Acc04343	228	26.04	7.64	42.64	86.40	-0.304	chloroplast	Mβ
AcMADS9	Acc04591	226	26.06	8.98	42.80	95.35	-0.616	cell nucleus	AP3
AcMADS10	Acc04856	229	25.50	5.32	55.68	84.8	-0.537	chloroplast	SVP
AcMADS11	Acc05140	164	19.12	7.72	35.27	80.79	-0.649	cytoplasm	Mγ
AcMADS12	Acc05042	208	24.51	8.93	56.41	72.64	-0.892	cytoplasm	AP3
AcMADS13	Acc05397	227	25.84	5.93	59.91	84.19	-0.728	cell nucleus	SVP
AcMADS14	Acc05562	242	28.08	5.95	51.64	75	-0.926	cell nucleus	FLC
AcMADS15	Acc06158	243	27.70	8.58	50.92	84.32	-0.612	cell nucleus	SEP
AcMADS16	Acc06870	218	24.66	9.17	35.03	75.96	-0.513	cell nucleus	Mα
AcMADS17	Acc07443	237	26.09	8.40	46.48	80.72	-0.332	chloroplast	Mα
AcMADS18	Acc07640	137	15.32	5.84	32.49	88.25	-0.378	cytoplasm	Mα
AcMADS19	Acc07652	215	24.91	9.23	51.41	86.19	-0.701	cell nucleus	SOC1
AcMADS20	Acc07718	162	18.66	9.89	56.45	89.69	-0.415	mitochondrion	Mγ
AcMADS21	Acc07950	226	25.85	6.33	65.10	82.83	-0.728	cell nucleus	SVP
AcMADS22	Acc08270	243	27.68	8.59	47.02	83.54	-0.623	cell nucleus	SEP
AcMADS23	Acc08300	310	35.83	6.83	39.25	67.00	-0.745	cell nucleus	Mγ
AcMADS24	Acc08919	499	56.40	7.09	37.62	70.52	-0.638	cell nucleus	Mδ
AcMADS25	Acc09079	249	28.64	8.55	49.97	81.04	-0.676	cell nucleus	AGL6
AcMADS26	Acc09279	248	28.10	9.12	54.63	84.07	-0.698	cell nucleus	AGL15
AcMADS27	Acc10230	235	27.09	9.01	51.31	88.34	-0.688	cell nucleus	ANR1
AcMADS28	Acc10522	227	25.83	8.35	58.10	80.31	-0.772	cell nucleus	SVP
AcMADS29	Acc10687	240	27.34	9.34	37.79	63.38	-0.713	cell nucleus	Mγ
AcMADS30	Acc11540	249	29.18	6.21	50.00	83.82	-0.814	cell nucleus	BS
AcMADS31	Acc12070	322	37.02	5.25	60.81	78.07	-0.672	cell nucleus	Mδ
AcMADS32	Acc00027	238	27.57	9.53	66.95	76.68	-0.813	cell nucleus	AG
AcMADS33	Acc13528	226	25.39	6.46	48.60	80.22	-0.644	cell nucleus	Mδ
AcMADS34	Acc13953	238	27.58	9.51	65.14	77.52	-0.824	cell nucleus	AG
AcMADS35	Acc14105	285	33.24	8.86	60.33	81.82	-0.742	cell nucleus	FUL
AcMADS36	Acc14300	245	28.08	8.81	51.58	80.04	-0.752	cell nucleus	SEP
AcMADS37	Acc14600	216	24.98	9.12	56.64	74.91	-0.881	cell nucleus	SOC1
AcMADS38	Acc14601	248	28.25	8.61	49.14	78.63	-0.649	cell nucleus	AGL6
AcMADS39	Acc15737	200	22.99	8.98	55.94	82.40	-0.830	cell nucleus	SOC1
AcMADS40	Acc16017	220	25.74	9.12	54.94	86.00	-0.760	cell nucleus	SOC1
AcMADS41	Acc16764	206	23.93	9.11	68.80	92.72	-0.683	cell nucleus	SOC1
AcMADS42	Acc17051	248	28.13	8.43	54.68	87.22	-0.613	cell nucleus	AGL15
AcMADS43	Acc17162	202	23.32	9.34	42.57	72.38	-0.737	mitochondrion	Mγ

AcMADS44	Acc18282	3<br>61	41.36	7.17	61.33	78.59	-0.620	cell nucleus	Mδ
AcMADS45	Acc18368	343	39.88	8.46	53.90	76.47	-0.852	cell nucleus	FUL
AcMADS46	Acc18807	242	27.53	9.17	35.50	66.07	-0.682	cell nucleus	Mγ
AcMADS47	Acc19959	200	23.27	7.76	59.38	85.80	-0.745	cell nucleus	SOC1
AcMADS48	Acc20728	248	28.58	9.4	52.81	75.52	-0.814	cell nucleus	AG
AcMADS49	Acc06844	238	27.28	9.39	61.67	97.06	-0.571	cell nucleus	ANR1
AcMADS50	Acc06819	238	27.28	9.78	53.77	86.01	-0.779	cell nucleus	ANR1
AcMADS51	Acc06804	209	24.28	9.85	54.70	89.09	-0.796	cell nucleus	SVP
AcMADS52	Acc21332	215	24.64	6.24	56.12	80.33	-0.737	cell nucleus	Mα
AcMADS53	Acc23601	226	26.16	9.14	43.02	88.01	-0.702	cell nucleus	AP3
AcMADS54	Acc23884	230	25.47	5.33	52.37	92.87	-0.406	chloroplast	SVP
AcMADS55	Acc24088	206	24.29	8.74	50.83	75.73	-0.849	cell nucleus	AP3
AcMADS56	Acc24142	201	23.07	8.41	46.89	92.69	-0.412	mitochondrion	AGL12
AcMADS57	Acc24145	224	25.69	9.40	54.56	90.62	-0.732	cell nucleus	AG
AcMADS58	Acc24404	195	21.51	6.65	38.90	68.00	-0.492	cytoplasm	Mα
AcMADS59	Acc24405	195	21.58	5.50	47.39	71.49	-0.343	cytoplasm	Mα
AcMADS60	Acc25178	248	28.58	9.18	51.71	80.65	-0.764	cell nucleus	AG
AcMADS61	Acc21883	245	27.94	8.69	51.44	86.41	-0.571	cell nucleus	SEP
AcMADS62	Acc22088	193	22.54	9.96	74.48	75.80	-0.758	cell nucleus	SOC1
AcMADS63	Acc26505	139	15.67	5.18	34.50	78.56	-0.619	mitochondrion	Mα
AcMADS64	Acc26639	238	27.68	9.47	67.99	81.64	-0.813	cell nucleus	FUL
AcMADS65	Acc26640	245	27.90	8.37	58.56	84.82	-0.578	cell nucleus	SEP
AcMADS66	Acc26835	162	18.82	10.20	62.34	75.25	-0.735	cell nucleus	SOC1
AcMADS67	Acc28717	298	32.49	4.59	37.50	62.18	-0.431	cytoplasm	Mα
AcMADS68	Acc28719	293	31.99	4.59	35.33	58.57	-0.522	cytoplasm	Mα
AcMADS69	Acc28871	209	24.12	9.41	71.55	92.78	-0.597	cell nucleus	SOC1
AcMADS70	Acc29299	247	28.44	8.84	50.48	84.53	-0.819	cell nucleus	FUL
AcMADS71	Acc29300	243	27.51	8.45	51.79	85.56	-0.587	cell nucleus	SEP
AcMADS72	Acc29875	357	40.98	6.54	61.75	78.94	-0.594	cell nucleus	Mδ
AcMADS73	Acc30912	190	20.94	9.17	36.48	74.95	-0.292	chloroplast	Mα
AcMADS74	Acc32725	245	28.04	8.81	50.76	78.45	-0.792	cell nucleus	SEP

Table S3 Protein conserved motif information of AcMADS-box

Motif	Sequence	Function
1	MGRGKVELKRIENKTNRQVTFSKRRNGLLKKAYELSVLCDAEVALIIFSP	MADS domain
2	KIENLQRSQRQLLGEDLGSLSIKELQQLERQLETSLKRIRSRKTQLMLEQ	K-box domain
3	RIENATNRQVTFSKRRNGLLKKAKELSVLCDAEVALIIFSPTGKLYEFAS	MADS domain
4	GKLYEFSSSSMMETI	K-box domain
5	IEELQKKEKALQEENEKLRKK	K-box domain
6	FFQPLDCNSTLQIGYNQVDPITVNAAG	unknown
7	AGGGGFWWDRPVGELGLGELERYMAALEELKCKVKSRADELAVAAVA EAM	unknown
8	DYGSDTGGAMVDPFCG	unknown
9	ERYQRCSYAERELNA	unknown
10	KKASSDESNTLSNSESNTQFYQQEAAKLR	Coiled-coil

Table S4 The Ka/Ks of tandem duplicate gene pairs in AcMADS-box family

Gene	Gene	Ka	Ks	Ka/Ks	Selective pressure
AcMADS1	AcMADS27	0.0600909	0.135317	0.444075	purify selection
AcMADS3	AcMADS6	0.0804497	0.170018	0.473183	purify selection
AcMADS4	AcMADS7	0.0371608	0.116784	0.3182	purify selection
AcMADS13	AcMADS21	0.0588794	0.121889	0.483056	purify selection
AcMADS13	AcMADS28	0.123799	0.661715	0.187088	purify selection
AcMADS15	AcMADS22	0.0220101	0.0667884	0.32955	purify selection
AcMADS21	AcMADS28	0.114209	0.761796	0.149921	purify selection
AcMADS32	AcMADS34	0.0240936	0.0865237	0.278463	purify selection
AcMADS36	AcMADS15	0.0592345	0.51865	0.114209	purify selection
AcMADS36	AcMADS22	0.0610039	0.487053	0.125251	purify selection
AcMADS36	AcMADS74	0.0180233	0.146346	0.123155	purify selection
AcMADS37	AcMADS19	1.02999	0.90187	1.14206	positive election
AcMADS37	AcMADS40	0.170193	0.762007	0.223348	purify selection
AcMADS38	AcMADS25	0.0496879	0.179568	0.276708	purify selection
AcMADS39	AcMADS47	0.0989295	0.163307	0.60579	purify selection
AcMADS40	AcMADS19	0.0515493	0.183503	0.280918	purify selection
AcMADS41	AcMADS69	0.0751541	0.166444	0.451527	purify selection
AcMADS42	AcMADS26	0.0546738	0.144077	0.379477	purify selection
AcMADS44	AcMADS72	0.050149	0.144051	0.348133	purify selection
AcMADS46	AcMADS29	0.0484004	0.241687	0.20026	purify selection
AcMADS47	AcMADS69	0.241009	0.76375	0.315559	purify selection
AcMADS48	AcMADS60	0.0310591	0.265419	0.117019	purify selection
AcMADS50	AcMADS49	0.0648263	0.114533	0.566006	purify selection
AcMADS53	AcMADS9	0.0511785	0.189419	0.270188	purify selection
AcMADS54	AcMADS10	0.0366683	0.171225	0.214152	purify selection
AcMADS55	AcMADS12	0.027621	0.254138	0.108685	purify selection
AcMADS59	AcMADS58	0.0498479	0.196806	0.253285	purify selection
AcMADS63	AcMADS18	0.0637257	0.2129038	0.29931	purify selection
AcMADS65	AcMADS61	0.15331	0.344643	0.444839	purify selection
AcMADS66	AcMADS62	0.94628	1.15508	0.819233	purify selection
AcMADS67	AcMADS68	0.095358	0.123574	0.771668	purify selection
AcMADS74	AcMADS15	0.0528937	0.529704	0.0998552	purify selection
AcMADS74	AcMADS22	0.0567536	0.480852	0.118027	purify selection

Table S5 The number of cis-acting elements in the promoter region of AcMADS-box gene family

Gene	Response type of cis-acting element								
	Optical	Auxin	Abscisic acid	Gibberellin	Salicylic acid	MeJA	Stress	Meristem	Circadian
AcMADS1	3	—	3	—	—	2	1	—	—
AcMADS2	4	—	2	—	—	2	1	—	—
AcMADS3	4	2	—	—	1	4	—	—	—
AcMADS4	5	2	5	—	—	2	1	—	1
AcMADS5	5	1	5	1	1	8	4	—	—
AcMADS6	1	2	—	—	—	—	3	—	—
AcMADS7	3	—	3	—	—	2	3	2	—
AcMADS8	1	1	—	—	—	2	1	—	—
AcMADS9	2	1	1	—	1	2	1	—	—
AcMADS10	1	—	—	—	1	—	3	—	—
AcMADS11	3	2	3	2	1	2	2	—	—
AcMADS12	5	—	3	1	—	—	4	—	—
AcMADS13	6	—	4	2	—	2	—	—	—
AcMADS14	—	—	—	1	—	—	3	—	—
AcMADS15	10	—	3	—	1	2	2	1	—
AcMADS16	—	—	—	—	—	2	3	—	—
AcMADS17	1	—	1	—	1	4	6	2	—
AcMADS18	1	—	—	2	1	2	2	—	—
AcMADS19	4	1	—	—	—	2	1	—	—
AcMADS20	2	—	1	1	2	—	3	—	—
AcMADS21	1	—	—	1	—	—	1	1	—
AcMADS22	3	—	1	—	1	2	1	—	1
AcMADS23	1	—	—	1	1	4	1	1	—
AcMADS24	2	1	—	1	—	2	2	—	—
AcMADS25	1	—	—	—	—	2	4	—	—
AcMADS26	1	—	—	—	1	2	2	—	1
AcMADS27	4	—	3	1	—	2	1	1	—
AcMADS28	—	—	—	—	—	—	—	—	—
AcMADS29	3	4	2	1	—	2	3	—	—
AcMADS30	3	1	1	—	1	2	4	—	—
AcMADS31	1	—	—	—	2	—	—	—	—
AcMADS32	3	—	1	—	—	—	3	1	—
AcMADS33	6	1	3	—	1	—	1	1	—
AcMADS34	3	—	1	—	—	4	—	1	—
AcMADS35	2	—	1	1	—	6	5	—	—
AcMADS36	1	—	—	—	—	—	3	—	1
AcMADS37	2	1	2	1	—	2	5	—	—

AcMADS38	3	1	3	1	—	—	2	—	—
AcMADS39	6	2	5	—		4	2	—	—
AcMADS40	3	—	—	—	—	—	6	1	—
AcMADS41	7	—	9	2	1	2	5	1	—
AcMADS42	1	4	2	1	—	—	5	—	1
AcMADS43	2	1	—	—	—	—	4	—	—
AcMADS44	—	1	—	—	—	2	1	—	1
AcMADS45	1	1	—	—	1	8	1	1	—
AcMADS46	2	—	1	2	1	6	2	—	—
AcMADS47	8	—	6	—	—	4	3	—	—
AcMADS48	1	4	—	1	—	2	2	2	—
AcMADS49	4	—	2	—	—	2	—	—	—
AcMADS50	—	—	—	1	1	—	3	1	—
AcMADS51	2	—	1	—	—	2	1	—	—
AcMADS52	—	—	—	—	1	—	2	—	—
AcMADS53	1	—	1	—	1	4	—	—	—
AcMADS54	2	1	1	—	—	—	4	—	1
AcMADS55	4	1	—	1	1	4	4	—	—
AcMADS56	2	1	—	1	—	—	2	1	1
AcMADS57	2	—	1	2	1	2	1	1	2
AcMADS58	4	—	1	1	1	4	3	—	—
AcMADS59	—	—	1	1	—	—	7	—	—
AcMADS60	—	1	—	—	1	—	1	—	1
AcMADS61	1	—	1	1	1	—	2	—	—
AcMADS62	—	1	—	1	1	2	2	—	—
AcMADS63	2	—	1	1	—	4	3	—	—
AcMADS64	3	—	1	—	—	—	1	—	—
AcMADS65	2	2	2	—	—	—	1	—	—
AcMADS66	3	—	1	1	2	—	1	—	—
AcMADS67	5	—	3	—	—	2	4	—	—
AcMADS68	5	—	2	—	—	—	4	—	—
AcMADS69	7	—	9	1	—	4	2	1	—
AcMADS70	1	—	2	1	—	6	4	1	—
AcMADS71	2	—	1	1	—	2	2	—	—
AcMADS72	4	—	—	—	2	2	6	—	—
AcMADS73	4	2	4	2	—	—	4	2	—
AcMADS74	7	—	4	—	—	—	2	—	—

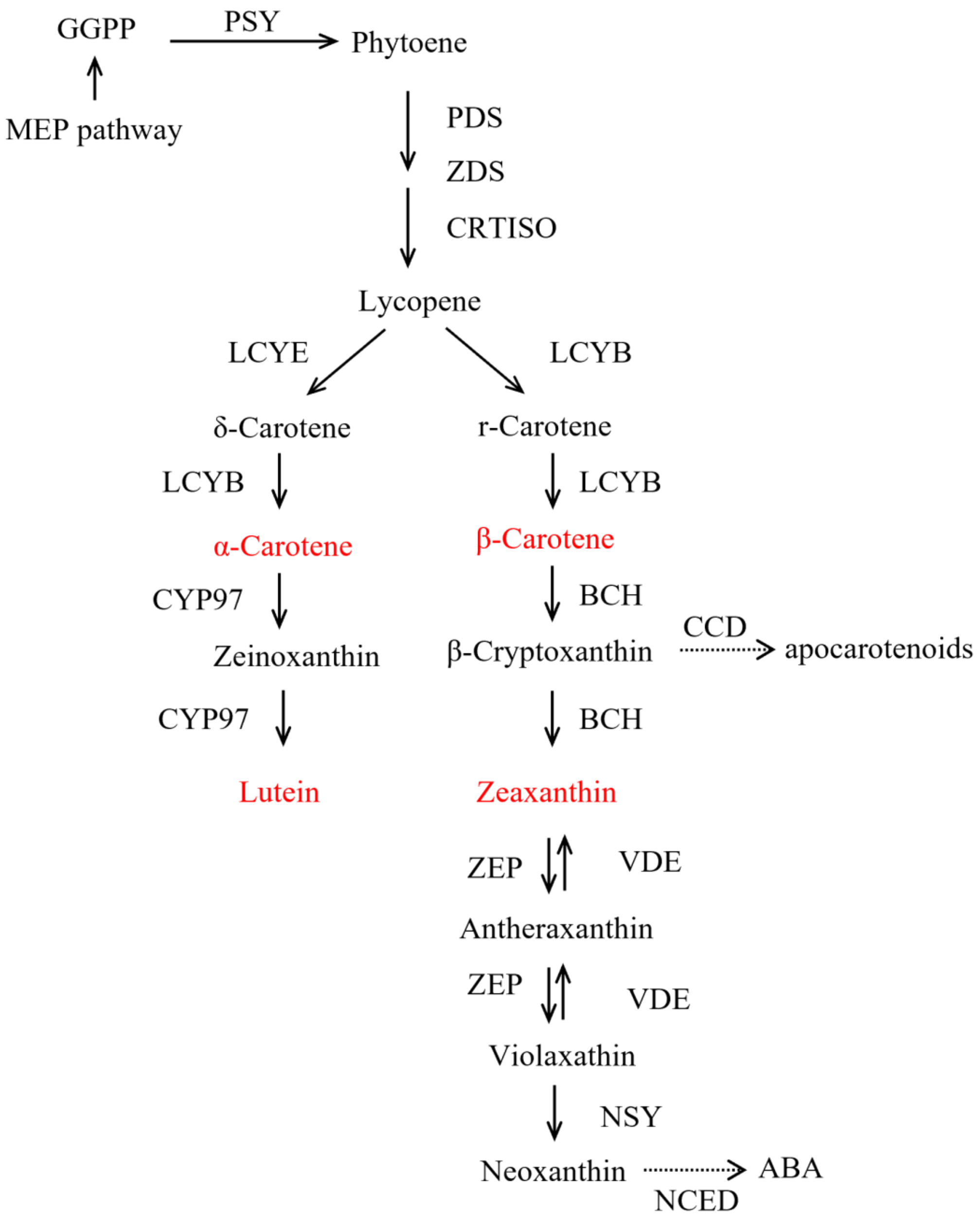

Fig. S1 Carotenoid biosynthesis pathway in higher plants.
